# When Antibiotics Are Refused: A Qualitative Study of Patient–Provider Communication, Trust, and Reported or Anticipated Responses in Jordan

**DOI:** 10.3390/healthcare14162612

**Published:** 2026-08-19

**Authors:** Mohammad Abu Assab, Anas Abed, Zekrayat J. H. Merdas, Mona Bustami, Saba Ammar Alabdali, Wafa’ A. Al-Haj, Wael Abu Dayyih, Sireen Abdul Rahim Shilbayeh, Zainab Zakaraya

**Affiliations:** 1 Clinical Pharmacy Department, Faculty of Pharmacy, Zarqa University, Zarqa 13110, Jordan; mabuassab@zu.edu.jo; 2 Department of Biopharmaceutics and Clinical Pharmacy, Faculty of Pharmacy, Al-Ahliyya Amman University, Amman 19328, Jordan; 202317079@ammanu.edu.jo (S.A.A.); z.zakaraya@ammanu.edu.jo (Z.Z.); 3 Department of Pharmacy, College of Pharmacy, Amman Arab University, Amman 11953, Jordan; th.merdas@aau.edu.jo; 4 Faculty of Pharmacy, University of Petra, Amman 11196, Jordan; mbustami@uop.edu.jo; 5 Faculty of Pharmacy, Al-Ahliyya Amman University, Amman 19328, Jordan; w.alhaj@ammanu.edu.jo; 6 Faculty of Pharmacy, Mutah University, Alkarak 61710, Jordan; wabudayyih@mutah.edu.jo; 7 Department of Pharmacy Practice, College of Pharmacy, Princess Nourah bint Abdulrahman University, P.O. Box 84428, Riyadh 11671, Saudi Arabia; ssabdulrahim@pnu.edu.sa

**Keywords:** antibiotic refusal, patient–provider communication, patient-centered care, trust, healthcare access, qualitative research, Jordan

## Abstract

**Highlights:**

**What are the main findings?**

- Antibiotic refusal was interpreted as a negotiation of credibility and care, not merely as denial of a requested medicine.
- Personally reported experiences, vignette-elicited intentions, and perceptions of other people’s behavior provided different forms of evidence and were analyzed separately.

**What are the implications of the main findings?**

- Non-antibiotic management may be more acceptable when providers acknowledge concerns, explain the reasoning, offer symptom-relief options, and provide safety-netting advice.
- Patient-centered communication should be supported by consistent prescribing and dispensing practices and accessible routes for clinical reassessment.

**Abstract:**

**Background/Objectives**: Patient-centered antimicrobial stewardship depends not only on restricting non-prescription antibiotic access, but also on how people interpret professional advice when an antibiotic request is declined. This study explored how adults in Jordan interpreted antibiotic refusal and distinguished personally reported experiences from anticipated vignette responses and perceptions of other people’s behavior. **Methods**: Semi-structured interviews were conducted between November 2025 and March 2026 with 32 adults purposively recruited for variation in age, gender, region, education, caregiving status, healthcare access, and antibiotic-use experience. Thirteen participants reported a previous refusal encounter—seven involving a pharmacist and six involving a physician—whereas 19 discussed refusal primarily through vignettes. Data were analyzed using an experiential, contextualist form of reflexive thematic analysis. **Results**: Three themes were developed: (1) antibiotics as active and responsible care under uncertainty; (2) refusal as a negotiation of credibility and care; and (3) continued antibiotic seeking after refusal as a set of reported, anticipated, or socially attributed possibilities. Antibiotics could symbolize strength, rapid recovery, and responsible caregiving. Refusal was more readily interpreted as care when participants described or anticipated acknowledgement, a reasoned explanation, symptom-relief options, and safety-netting. Responses after refusal included acceptance and monitoring, uncertainty or clinical reassessment, and continued antibiotic seeking, but these were not equivalent forms of evidence. **Conclusions**: Antibiotic refusal is a trust-sensitive and context-dependent patient–provider interaction rather than a uniform behavioral pathway. The findings identify communication and access conditions that merit prospective evaluation; they do not establish that communication causes acceptance or that vignette intentions predict behavior.

## 1. Introduction

Antimicrobial resistance (AMR) is a major public-health threat that compromises the treatment of common bacterial infections. In 2019, bacterial AMR was estimated to have caused 1.27 million deaths directly and to have been associated with 4.95 million deaths worldwide [1]. Inappropriate antibiotic use in community settings is an important modifiable contributor, including use without clinical assessment, use for self-limiting illness, premature discontinuation, sharing, and storage for later use [2].

Community pharmacies and primary-care services are central to antibiotic stewardship because many decisions about acute symptoms occur outside hospitals [3]. In low- and middle-income settings, pharmacies provide rapid access to advice and treatment, but inconsistent enforcement, patient demand, and commercial pressures may also facilitate non-prescription supply [4]. In Jordan, simulated-patient and mixed-methods studies have documented continued non-prescription antibiotic dispensing despite regulatory restrictions [5,6,7]. Population studies have also identified misconceptions about antibiotics, resistance, and self-medication [8,9,10].

Antibiotic stewardship in community care is also a patient–provider communication issue. Decisions are shaped by prior treatment experiences, perceived illness severity, professional authority, and consultation dynamics [11,12,13,14,15,16]. Non-antibiotic management may be acceptable when providers offer a credible explanation, practical symptom relief, reassurance, and safety-netting [11,15,16,17]. These components are consistent with patient-centered care but do not establish that any single communication strategy will reliably change behavior across settings.

Jordanian research has quantified public knowledge and antibiotic-use practices, documented non-prescription antibiotic dispensing, and examined antimicrobial stewardship from healthcare professionals’ perspectives. Recent realist-informed qualitative research in Jordanian hospitals also explored how organizational structures, interprofessional relationships, workflow integration, and prescribing cultures shape clinical pharmacists’ contributions to team-based antimicrobial stewardship [18]. However, this provider- and hospital-centered literature does not explain how lay adults interpret antibiotic refusal in community-facing encounters or what they report, anticipate, or believe others might do afterwards. These evidentiary sources should not be treated as equivalent: a personally recalled action has a different status from an intention expressed in response to a vignette or a generalized perception of community behavior.

Jordan’s National AMR Monitoring and Evaluation Plan for 2023–2025 prioritizes awareness, rational antimicrobial use, surveillance, infection prevention, research, and multisectoral coordination [19]. Examining how refusal is interpreted can inform these priorities and patient-centered engagement in community care.

This study aimed to explore how adults in Jordan interpreted antibiotic refusal by pharmacists and physicians and how previous experience, social context, professional authority, communication, and healthcare access shaped reported or anticipated responses. A secondary analytic objective was to distinguish personal refusal experiences from vignette-based intentions and perceptions of other people’s behavior.

## 2. Materials and Methods

### 2.1. Study Design, Orientation, and Reporting

This exploratory qualitative study used one-to-one semi-structured interviews to examine meanings, experiences, and anticipated responses surrounding antibiotic refusal. Reporting followed the Consolidated Criteria for Reporting Qualitative Research (COREQ) [20]. The completed COREQ checklist is provided as Supplementary Material S1.

The analysis was situated within a contextualist, critical-realist position. Participant accounts were treated as meaningful but situated descriptions of experience, shaped by social norms, memory, professional roles, the interview context, and vignette prompts. Critical realism was selected because it supports analysis of both subjective meaning-making and the material or structural conditions that shape it, including professional authority, regulatory consistency, and healthcare access [21]. Comparable qualitative health research has used this position to examine how sociocultural and health-system conditions influence communication and shared decision-making in non-Western settings [22]. The analytic orientation was primarily experiential: coding attended mainly to semantic content and, where supported, to latent meanings such as antibiotics symbolizing responsible care or refusal functioning as a test of credibility.

### 2.2. Study Setting

The study was conducted in Jordan between November 2025 and March 2026. Community pharmacies are widely accessible and frequently serve as a first point of contact for minor acute symptoms. Interviews were conducted remotely by telephone or online video call, according to participant preference, to facilitate participation across governorates and reduce geographical barriers. Before each interview, participants confirmed that they were in a private setting where they could speak freely and without interruption.

### 2.3. Research Team and Reflexivity

The research team comprised academic pharmacists and researchers with experience in pharmacy practice, antimicrobial stewardship, public health, and qualitative research. Interviews were conducted by A.A., a male academic clinical pharmacist with postgraduate research training and previous qualitative interviewing experience. The interviewer had no prior personal or professional relationship with participants.

Because several researchers were pharmacists, the team reflected on the risk of privileging stewardship interpretations or treating refusal as inherently correct. The interview guide invited disagreement, frustration, and distrust; the interviewer used neutral prompts and did not correct participants. Reflexive memos considered how professional assumptions, vignette wording, and the team’s policy interests shaped data generation and theme development.

### 2.4. Participants and Eligibility

Participants were eligible if they were aged 18 years or older, residing in Jordan, able to communicate in Arabic, willing to provide informed consent, and willing to participate in an audio-recorded interview. Healthcare professionals and students in health-related disciplines were excluded to focus on lay perspectives. Prior refusal experience was not required because the study intentionally included both experiential narratives and vignette-based responses.

### 2.5. Sampling and Sample Sufficiency

Purposive maximum-variation sampling sought diversity in age, gender, educational level, marital and caregiving status, region and type of residence, health-insurance coverage, previous non-prescription antibiotic use, and usual first point of care. The sample was deliberately enriched with people who had relevant antibiotic-related experiences, including prior non-prescription use, refusal by a pharmacist or physician, use of leftovers, caregiving responsibilities, and reliance on community pharmacies. The sample was designed for interpretive depth and was not statistically representative of the Jordanian population.

Screening characteristics were recorded in a recruitment matrix and reviewed periodically. Later recruitment gave particular attention to older adults, people outside central urban areas, participants with lower educational attainment, those without health insurance, and those without previous non-prescription antibiotic use. Recruitment decisions also considered underrepresented experiences, especially pharmacist or physician refusal, caregiving, and different patterns of healthcare access. These efforts aimed to broaden perspectives rather than achieve demographic quotas.

Forty-three individuals expressed interest and 39 completed eligibility screening; reasons why four did not complete screening were not recorded. Three screened individuals were ineligible (two health-related students and one person working in a healthcare-related setting). Thirty-six were eligible and invited; 32 completed interviews. Two could not be scheduled after repeated contact attempts, one cancelled because of time constraints, and one was lost to follow-up. No participant withdrew after an interview began.

Sample sufficiency was considered using information power [23], based on the focused aim, relevance and diversity of participants, depth of dialogue, and adequacy of the dataset for developing a coherent interpretive account. The final sample reflected the planned qualitative design rather than substantial non-response: 32 of 36 eligible invitees completed interviews. Recruitment concluded when the dataset supported stable central organizing concepts, source-specific comparison, and contrasting cases, while recognizing that further interviews might have generated additional meanings. Information power was therefore not treated as a numerical saturation threshold, and the findings were not intended to estimate national prevalence.

### 2.6. Recruitment

A standardized invitation was circulated through WhatsApp, Facebook, community groups, and personal networks of non-healthcare contacts in different Jordanian governorates. Researchers and intermediaries could forward the invitation to acquaintances who might meet the eligibility criteria, but participation was voluntary and no person was nominated or pressured to take part.

Interested individuals completed a Google Forms screening form or provided the same information by telephone or WhatsApp. The screening form was used only for eligibility and maximum-variation sampling and was not analyzed as qualitative data. Eligible participants received the information sheet, had an opportunity to ask questions, and were contacted by the interviewer to arrange the interview.

Non-healthcare contacts and community intermediaries only redistributed the invitation. They did not screen, consent, schedule, or interview participants and had no access to participant responses. The interviewer had no prior relationship with participants. Where another researcher or intermediary knew a potential participant socially, that person was not involved in screening, consent, interviewing, or analysis of that participant’s account. No financial or material compensation was provided.

### 2.7. Interview Guide and Vignette Design

The guide explored healthcare seeking, previous antibiotic use, treatment expectations, personal experiences of refusal, trust in pharmacists and physicians, possible sources of antibiotics, and communication preferences. Participants were first invited to provide spontaneous personal narratives. Vignettes were introduced later to explore situations not personally experienced. During transcription and analysis, vignette-elicited responses were distinguished from spontaneous accounts. The full semi-structured interview guide and vignette prompts are provided as Supplementary Material S2.

The exact vignette wording is reported in Table 1. The scenarios were designed to explore interpretations of provider advice, not to diagnose infection from nonspecific symptoms. The communication-contrast vignette explicitly stated that the illness appeared viral, which may have signaled a preferred response. The child vignette did not specify age or risk profile, and pharmacist- and physician-refusal scenarios were not identical. Responses therefore cannot be treated as controlled comparisons of provider type or communication components.

### 2.8. Data Management, Transcription, and Translation

Audio recordings were transcribed verbatim in Arabic and checked against the recordings. Identifying information, including names, workplaces, telephone numbers, and specific locations, was removed, and each participant was assigned a study code.

Analysis was conducted primarily in Arabic to preserve local meanings and colloquial phrasing. Extracts selected for publication were translated into English by one bilingual researcher and independently checked by a second for conceptual fidelity. Minor readability edits preserved meaning, tone, hesitation, uncertainty, and contradiction.

Recordings, de-identified transcripts, dated verbal-consent logs, field notes, and analytic files were stored on password-protected devices and secure institutional cloud storage accessible only to authorized team members. Audio files were deleted after transcript verification. De-identified transcripts and analytic records will be retained for five years in accordance with institutional practice.

### 2.9. Reflexive Thematic Analysis

Data were analyzed using Braun and Clarke’s reflexive thematic analysis [24,25,26]. The process involved iterative familiarization, coding, development and review of candidate themes, refinement of boundaries and central organizing concepts, and production of the analytic account. The analysis was data-led but not fully inductive; it was sensitized by the interview guide and literature on antibiotic expectations, trust, access, and stewardship. Coding combined semantic attention to explicit statements with selective latent interpretation of meanings attached to strength, responsibility, authority, and care.

A.A. led coding and theme development. A second researcher read six early transcripts and participated in collaborative reflexive discussions. The purpose was not independent verification, consensus coding, or resolution of disagreement. Different readings were treated as interpretive resources that prompted reconsideration of assumptions, code boundaries, and alternative thematic constructions. The wider team discussed developing themes and deviant cases, while the lead analyst retained responsibility for final interpretive decisions.

No inter-coder reliability statistic was calculated or sought. NVivo 14 supported data organization and retrieval. Analytic memos and evolving thematic maps documented theme development rather than functioning as a fixed codebook. The final coding tree and analytic development record are provided as Supplementary Material S3.

Coded extracts were indexed by evidentiary source—personal experience, vignette response, observation of another person, or generalized perception—and by relevant contextual characteristics. This indexing informed the source-specific reporting and qualitative comparisons in Section 3.

### 2.10. Trustworthiness and Participant Follow-Up

Credibility was supported through open-ended interviewing, maximum-variation sampling, exploration of personal experience before vignette prompting, attention to contrasting and deviant cases, source-specific reporting, and contextually informative extracts. Reflexive memos and team discussions documented how professional backgrounds and vignette wording may have influenced data generation and interpretation.

Field notes informed familiarization and subsequent probing but were not analyzed as an independent dataset. Full transcripts were not returned to participants. A brief Arabic summary of preliminary findings was shared with six participants who had agreed to further contact. Their responses were used as feedback on resonance and clarity, not as validation of a single objectively correct interpretation. Feedback clarified wording but did not determine the thematic structure.

### 2.11. Ethical Considerations

Ethical approval was obtained from the Institutional Review Board of Al-Ahliyya Amman University before recruitment commenced (AAU/PHARM/1/2/1/2025–2026; approved 17 October 2025). All procedures followed the approved protocol and relevant institutional requirements.

Eligible participants received an information sheet describing the study purpose, voluntary participation, interview procedures, audio recording, confidentiality, use of anonymized quotations, and withdrawal rights. Before each remote interview, participants had an opportunity to ask questions and provided recorded verbal informed consent, which was documented in a dated consent log under the IRB-approved remote-consent procedure.

Participants could decline any question, stop the interview, or request withdrawal of their data within the period specified in the information sheet. Quotations used participant codes and limited contextual descriptors to minimize identification risk. No financial or material compensation was provided.

## 3. Results

### 3.1. Participant Characteristics

Thirty-two adults were interviewed. The sample was purposively enriched for antibiotic-related experience and was not intended to represent the Jordanian population. Participants were predominantly urban and relatively highly educated; 22 (68.8%) reported previous non-prescription antibiotic use. Thirteen participants reported a prior refusal of antibiotics: seven by a pharmacist and six by a physician. Nineteen had no previous refusal experience and discussed refusal mainly through vignette prompts. Participant characteristics are summarized in Table 2.

### 3.2. Evidentiary Sources and Analytic Structure

The analysis distinguished four sources of evidence: personally reported experiences, anticipated responses elicited through vignettes, observations concerning family members or other people, and generalized perceptions of community behavior. Personally experienced actions are reported as participant-reported behavior; vignette responses are presented as anticipated responses; and third-person accounts are interpreted as perceptions rather than documented personal actions.

Because participants could contribute more than one type of account, the evidence-source categories were not mutually exclusive at the interview level. Qualitative frequency terms are used descriptively and do not indicate prevalence. Participants with prior refusal experience provided more behaviorally grounded accounts, whereas those without such experience generally described conditional anticipated responses.

Three themes were developed around central organizing concepts rather than the interview-guide topics alone. Table 3 summarizes each theme and the principal evidence supporting it.

### 3.3. Theme 1: Antibiotics as Active and Responsible Care Under Uncertainty

For some participants, antibiotics represented an active response to uncertainty, associated with strength, rapid recovery, and resuming work or caregiving. Previous prescriptions gave these expectations experiential legitimacy, while family networks circulated antibiotic names and recovery stories as practical knowledge. In child-related accounts, seeking “something strong” could signify responsible caregiving rather than certainty about bacterial infection.

Previous prescriptions were often used as templates for later illness episodes:

“*The first time I had this throat pain, the doctor prescribed an antibiotic and I improved quickly. So when it happened again, I went to the pharmacy already thinking of the same medicine. I knew it might not be exactly the same illness, but I had work the next morning and waiting felt like taking a risk.*”

(P11, female, 29 years; reported personal experience; previous non-prescription antibiotic use)

The expected benefit reflected pressure for rapid recovery rather than certainty that the later illness was clinically identical. Improvement without antibiotics provided a competing experience. A contrasting participant who had not previously used non-prescription antibiotics described symptom management and clinical examination as the more active response, indicating that decisiveness was not uniformly equated with antibiotic use.

Parenting intensified the perceived responsibility to act, although anticipated antibiotic seeking remained conditional:

“*My first reaction in the child situation is that I would want an antibiotic because a child’s fever makes me nervous. For myself I can wait, but with my son I feel responsible if I delay. Still, if the pharmacist told me what temperature or symptoms were dangerous, how long the illness should last, and when to take him to a doctor, I think I could wait instead of searching elsewhere.*”

(P20, female, 36 years, parent; vignette-elicited anticipated response)

Together, these accounts show that antibiotics could carry meanings of decisiveness, speed, and responsible caregiving, but those meanings were neither fixed nor universal. They could be reconsidered through improvement without antibiotics, preference for examination, or clear information about expected illness duration and warning signs.

### 3.4. Theme 2: Refusal as a Negotiation of Credibility and Care

Antibiotic refusal became a moment in which participants evaluated professional credibility and whether their illness had been taken seriously. Acknowledgement, explanation, practical symptom-management advice, and a plan for worsening symptoms signaled active care; a brief refusal could instead appear rule-based, unsupported, or dismissive.

One participant reported that a pharmacist asked about symptom duration and breathing difficulty, recommended paracetamol, and provided safety-netting. These actions changed an initially rule-based interpretation of refusal. Augmentin is a brand name for amoxicillin/clavulanate:

“*Because he explained and did not dismiss me, I went home and did not try another pharmacy. I improved after two days.*”

(P07, male, 32 years; reported personal experience of pharmacist refusal)

Provider identity also shaped initial credibility. Physicians were commonly associated with diagnostic examination, while pharmacists were valued for accessibility and medicines expertise. One participant who experienced physician refusal reported that examination and explanation increased trust, although uncertainty remained until the fever subsided. Because the scenarios were not identical and most participants had not experienced both forms of refusal, this pattern reflects social understandings of professional roles rather than a controlled comparison.

Some accounts resisted a simple acceptance-versus-resistance distinction. A participant who understood the pharmacist’s explanation still sought medical review because symptoms remained disruptive and she feared that something had been missed:

“*The pharmacist gave a detailed explanation … and I understood what he meant. But I had already been sick for four days and could not sleep, so the next morning I went to a clinic. I was not going only to obtain an antibiotic; I wanted someone to examine me.*”

(P18, female, 42 years; reported pharmacist-refusal experience followed by clinical reassessment)

Seeking further care therefore did not necessarily mean attempting to override stewardship advice. Refusal was negotiated through professional authority, previous treatment experience, symptom progression, and whether the encounter was experienced as active care.

### 3.5. Theme 3: Continued Antibiotic Seeking After Refusal—Reported, Anticipated, and Socially Attributed Possibilities

The author-generated label “continued antibiotic seeking after refusal” was used instead of “antibiotic shopping,” which was not an in vivo term and could be stigmatizing. Participants discussed visiting another pharmacy, seeking medical assessment, using stored antibiotics, requesting a medicine by name, or renewing pressure; these possibilities were not treated as equivalent documented behavior.

The strongest evidence came from personally reported actions after an actual refusal:

“*The first pharmacist refused because I did not have a prescription. … I was convinced that the same antibiotic had helped me before, so I went to another pharmacy near my home. They supplied it after I repeated that I had used it previously.*”

(P05, female, 27 years; reported pharmacist refusal followed by continued antibiotic seeking)

This account shows how inconsistent dispensing could make refusal appear to be a local procedural barrier rather than a shared professional standard. Participants without prior refusal experience more often described conditional intentions:

“*If the pharmacist explained and gave me something for the symptoms, I think I would wait. But if … the fever continued, I might ask another pharmacy or see a doctor.*”

(P16, male, 24 years; vignette-elicited anticipated response; no previous refusal experience)

Third-person accounts were reported as perceived social norms rather than personal behavior. For example, one participant described a brother who visited multiple pharmacies because professional opinions appeared inconsistent, while explicitly stating that she had not done so herself (P25, female, 51 years).

A contrasting account distinguished legitimate reassessment from searching for a willing prescriber:

“*Once the doctor has examined me and says an antibiotic is not needed, I do not search for someone who will prescribe it. If I remain worried, I would return for another assessment.*”

(P30, male, 57 years; vignette-elicited response informed by previous clinical experience)

Refusal therefore did not produce one uniform response. The meaning of subsequent help-seeking depended on whether the account concerned reported behavior, anticipated intentions, perceived social norms, or clinically appropriate reassessment.

### 3.6. Comparison by Prior Refusal Experience and Participant Context

The 13 participants with prior refusal experience provided more concrete interactional detail than the 19 who responded primarily to vignettes, including what the provider assessed, how the decision was explained, what alternatives were offered, and what occurred afterwards. Vignette-only participants more often used conditional language linked to symptom persistence, work or study obligations, concern for a child, access to a clinic, and whether an alternative plan was offered.

Across both groups, explanation, symptom management, and safety-netting indicated that the illness had been taken seriously. Actual-experience accounts showed that acceptance could coexist with anxiety or appropriate reassessment. Previous non-prescription use made familiar names and past recovery more salient for some participants but did not determine response. Parenting intensified uncertainty, while cost, travel, waiting time, and work constraints sometimes made another pharmacy appear more feasible than a clinic. Patterns by education or region were insufficiently coherent for strong interpretation.

### 3.7. Proposed Interpretive Model

**Figure 1:**
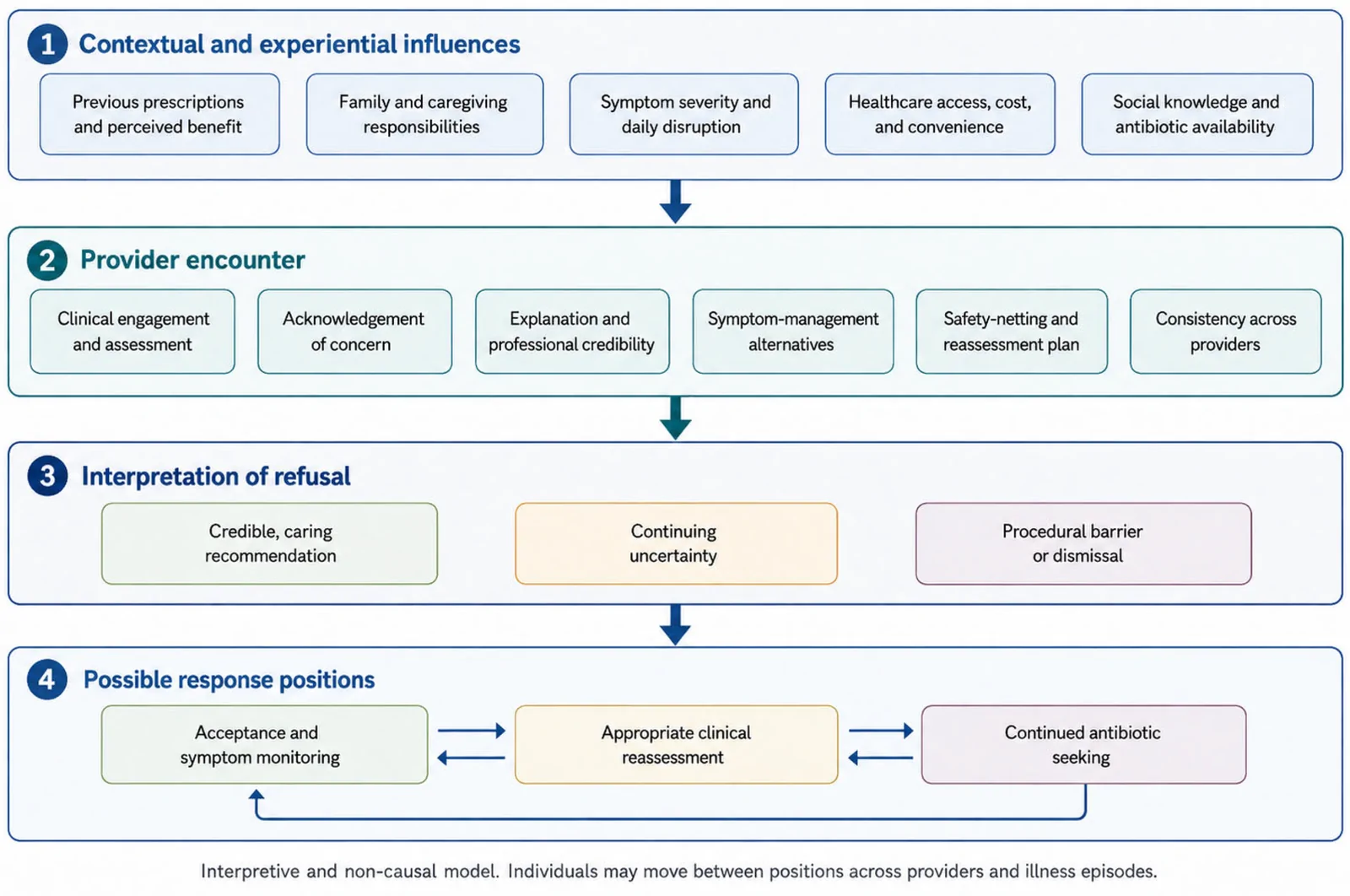
Layered interpretive model of responses to antibiotic refusal. Pre-encounter influences and features of the provider encounter shape whether refusal is interpreted as credible active care, unresolved uncertainty, or a procedural barrier/dismissal. These interpretations are linked to possible response positions. Relationships are interpretive and non-causal; responses may change as symptoms evolve and may inform future expectations.

Figure 1 integrates four interacting layers: pre-encounter influences, the provider encounter, interpretation of refusal, and possible response positions. Prior experiences, illness and caregiving pressures, healthcare access, system consistency, communication, and professional authority jointly shaped how refusal was interpreted.

The response positions—acceptance and monitoring, clinical reassessment, and continued antibiotic seeking—are not fixed, mutually exclusive, sequential, or causal. Individuals may move between them as symptoms evolve, and a later experience may become part of the prior-experience context for a future illness episode.

## 4. Discussion

This study examined antibiotic refusal as a patient–provider interaction rather than only a medicine-supply decision. Its principal contribution is to show how credibility, care, and uncertainty were negotiated while separating reported experiences, vignette intentions, and perceptions of others. Antibiotics could signify active care; refusal was more credible when coupled with acknowledgement, explanation, symptom management, and safety-netting; and responses ranged from acceptance to reassessment or continued antibiotic seeking. These findings extend Jordanian research beyond knowledge and dispensing rates by explaining how non-antibiotic care is interpreted [5,6,7,8,9,10,14].

### 4.1. Antibiotics as Active Care Under Uncertainty

Antibiotic expectations were not reducible to confusion about bacterial and viral infection. Participants linked antibiotics with strength, speed, and resuming work or caregiving, so requesting them could represent action under uncertainty. This helps explain why knowledge and antibiotic expectation may coexist when illness is disruptive or emotionally salient [8,9,10].

Previous prescriptions, perceived past benefit, familiar names, and social learning served as experiential authority [13,14,27]. A previous prescription may become a template for later episodes, making a clinically appropriate refusal appear inconsistent. Public communication should therefore address why recovery after antibiotic use does not necessarily establish benefit in a self-limiting illness, rather than relying only on general resistance messages.

Child-related accounts showed that antibiotic requests could also express protective responsibility. Similar Jordanian evidence identifies gaps in parental antibiotic knowledge and practices [28], but the present findings additionally highlight anxiety and fear of delayed action. A patient-centered response can validate concern while offering active alternatives: symptom relief, expected-duration advice, warning signs, and clear thresholds for review.

### 4.2. Refusal as a Negotiation of Credibility and Care

Participants assessed refusal through its clinical content and relational delivery. Symptom questions, examination or referral, practical alternatives, and safety-netting signaled engagement rather than simple denial.

This interpretation aligns with evidence that prescribing is shaped by perceived demand, consultation dynamics, trust, and satisfaction [12,15,29], and that non-antibiotic management is more acceptable when accompanied by a positive explanation, reassurance, and follow-up advice [11,16]. The present study frames these components as evidence of care.

These findings do not establish causal effectiveness. The communication vignette varied several components and explicitly suggested a viral illness, while actual accounts showed that understanding could coexist with residual worry or later reassessment. Patient-centered communication should therefore allow uncertainty and preserve access to review rather than equating acceptance with complete reassurance.

Provider identity shaped initial credibility: physicians were associated with diagnosis and pharmacists with access and medicines expertise. This was not a controlled comparison, but it is relevant where community pharmacies are first-contact services and non-prescription dispensing varies [5,6,7]. A pharmacist’s relational communication may be weakened if another provider supplies the requested antibiotic, underscoring the need for consistent system support.

### 4.3. Responses After Refusal and the Role of Healthcare Access

Refusal did not always end antibiotic seeking, but the evidentiary source determined what could be claimed. Reported actions, conditional vignette intentions, and third-person perceptions were analyzed separately; this distinction is relevant to other qualitative vignette studies of healthcare behavior.

Some reported visits to another pharmacy suggested that inconsistent dispensing could turn refusal into a temporary obstacle. International evidence similarly links non-prescription supply to patient demand, regulatory implementation, commercial pressures, and provider practice [5,30,31]. However, subsequent consultation could represent appropriate reassessment rather than bypassing advice. Stored antibiotics also created access outside the encounter, consistent with evidence on leftovers and sharing [13,14,32].

Cost, transport, waiting time, and work constraints shaped whether another pharmacy or a clinic appeared feasible [13,27,33]. Communication is therefore inseparable from timely and affordable assessment; safety-netting is less actionable when access barriers remain.

### 4.4. Implications for Patient-Centered Communication and Community Stewardship

The interpretive model positions acceptance, clinical reassessment, and continued antibiotic seeking as changeable rather than linear responses. A person may accept advice initially, seek review if symptoms persist, and revise expectations after conflicting advice.

Practical communication training should be role-specific. Pharmacists should be supported to conduct brief symptom and red-flag assessment, acknowledge concern, explain why an antibiotic is not currently indicated, recommend feasible symptom relief, and provide explicit safety-netting. Physicians should address how previous prescriptions shape current expectations, distinguish the present illness from earlier episodes, and explain the expected course and reassessment threshold. Across professions, shared wording and referral pathways could reduce contradictory messages.

At the policy level, public communication should normalize non-antibiotic management as active care, while regulators and professional bodies support consistent prescription-only practice, accessible clinical reassessment, and audit or feedback on dispensing. Delayed prescribing may be appropriate for selected primary-care respiratory infections after clinical assessment [11,34], but it should not be conflated with pharmacist refusal. The proposed communication components require prospective evaluation rather than being treated as a validated script.

### 4.5. Transferability Beyond Jordan

Several interpretive patterns may transfer to other middle-income systems where community pharmacies are accessible first-contact services, non-prescription antibiotic access varies, and medical consultation involves financial, geographic, or occupational barriers. Studies and reviews from Iraq, the Middle East, and other low- and middle-income settings similarly identify convenience, prior experience, household availability, and inconsistent provider practices as influences on antibiotic use [21]. However, transferability should be judged against pharmacy regulation, primary-care accessibility, professional roles, and cultural expectations of treatment. In systems with strict prescription enforcement and accessible primary care, bypassing refusal may be less feasible, although trust, prior prescribing, and relational communication may remain relevant.

## 5. Strengths and Limitations

This study addresses a locally relevant gap by examining the meaning of antibiotic refusal rather than measuring knowledge or use alone. Combining spontaneous narratives with vignettes enabled participation by adults with and without prior refusal experience. Distinguishing reported experiences, vignette intentions, observations of others, and generalized perceptions increased analytic transparency. Other strengths included maximum-variation sampling, analysis in Arabic, reflexive engagement, field notes and analytic memos, and attention to contrasting cases.

Several limitations constrain interpretation. The purposive sample was enriched for antibiotic-related experience, predominantly urban, and relatively highly educated; it was not nationally representative. This composition may have favored adults who were more familiar with navigating healthcare, more able to articulate provider interactions, or more able to access multiple care options, while underrepresenting rural, less-educated, or digitally disconnected adults whose trust, access constraints, and responses may differ. Although 32 interviews provided sufficient information power for the focused qualitative aims, only 13 participants reported an actual refusal and 19 discussed refusal primarily through vignettes. The study therefore does not estimate national prevalence, and anticipated actions or perceptions of others may differ from behavior during illness.

The vignettes contained nonspecific symptoms, omitted the child’s age and risk profile, and were not identical across provider types. The communication contrast explicitly suggested a viral illness and varied several communication components simultaneously. These features may have shaped responses and prevent attribution of preference to any single element.

Recall and social desirability may have influenced accounts. Remote interviewing limited observation of nonverbal communication, although it enabled participation across governorates. The cross-sectional design cannot establish a stable sequence or causal effect of communication. The interpretive model represents possible positions rather than a confirmed pathway.

Contextual comparisons involving previous non-prescription antibiotic use, parenting, healthcare access, region, and education were exploratory. They were interpreted only where several accounts supported a coherent pattern and should not be read as demographic associations.

## 6. Conclusions

Antibiotic refusal was a trust-sensitive and context-dependent patient–provider interaction. Participants interpreted it in relation to previous prescribing, social advice, illness burden, caregiving responsibility, healthcare access, professional authority, and whether the encounter demonstrated acknowledgement and active care.

Possible positions after refusal included acceptance and monitoring, continued uncertainty or clinical reassessment, and continued antibiotic seeking after refusal. These findings comprised personally reported actions, vignette-elicited intentions, and perceptions of others and should not be treated as equivalent documented behavior.

Participants valued reasoned explanations, practical symptom-management options, acknowledgement of concern, and clear safety-netting because these elements made non-antibiotic management visible as care. Community stewardship should combine patient-centered communication with consistent prescription-only practice and accessible reassessment. Prospective observational and intervention studies are needed to determine whether specific communication approaches change behavior in routine care.

## Figures and Tables

**Table 1:** Vignette wording and interpretive cautions.

Vignette	Wording Used in the Interview Guide	Interpretive Caution
1. Adult/pharmacist	“Imagine that you have had a sore throat, cough, runny nose, and mild fever for two days. You go to a community pharmacy and ask for an antibiotic. The pharmacist says that antibiotics are not needed and recommends symptomatic treatment instead. How would you feel? Would you accept the pharmacist’s advice? What would you do next?”	The symptoms are nonspecific. Responses are hypothetical intentions and should not be reported as documented behavior.
2. Child/pharmacist	“Imagine that your child has a sore throat, cough, and fever for two days. The pharmacist says antibiotics are not appropriate at this stage and advises fever control, fluids, and medical review if symptoms worsen or continue. Would your response be different because the patient is a child?”	The child’s age, comorbidities, and clinical-risk profile were not specified; participants may have imagined different pediatric contexts.
3. Previous prescription	“Imagine that you had similar symptoms last year and a doctor prescribed an antibiotic. This time, a pharmacist or physician says that antibiotics are not needed. How would you interpret this difference?”	The vignette explores how previous prescribing shapes expectations. Pharmacist and physician refusal are combined and should not be treated as an experimental comparison.
4. Communication contrast	Provider A states: “No, you do not need antibiotics.” Provider B acknowledges the wish to recover, explains that antibiotics are unlikely to help because the illness appears viral, recommends symptomatic treatment, and provides warning signs and advice about when to seek further care. Participants were asked which response they trusted more and why.	The second response varies empathy, explanation, alternatives, safety-netting, and diagnostic framing simultaneously. Preference cannot be attributed to any single component.

Note: The guide was piloted with three adults to assess clarity, cultural appropriateness, sequencing, and vignette wording. Pilot interviews were excluded after minor wording revisions. No repeat interviews were conducted. Interviews were conducted in Arabic, lasted approximately 30–45 min, and were audio-recorded with permission. The interviewer completed field notes immediately after each interview.

**Table 2:** Sociodemographic and antibiotic-use characteristics of participants (*n* = 32).

Characteristic	Category	*n* (%)
Age group	18–29 years	9 (28.1)
	30–44 years	12 (37.5)
	45–59 years	8 (25.0)
	≥60 years	3 (9.4)
Gender	Female	18 (56.3)
	Male	14 (43.7)
Region of residence	Central Jordan	18 (56.3)
	Northern Jordan	8 (25.0)
	Southern Jordan	6 (18.7)
Type of residence	Urban	23 (71.9)
	Semi-urban/rural	9 (28.1)
Educational level	Secondary education or less	7 (21.9)
	Diploma	6 (18.7)
	Bachelor’s degree	15 (46.9)
	Postgraduate degree	4 (12.5)
Employment status	Employed	17 (53.1)
	Unemployed/household work	8 (25.0)
	Student	4 (12.5)
	Retired	3 (9.4)
Marital status	Married	21 (65.6)
	Single	9 (28.1)
	Divorced/widowed	2 (6.3)
Parent/caregiver status	Has children or regularly cares for children	20 (62.5)
	No children/caregiving role	12 (37.5)
Health insurance coverage	Public insurance	13 (40.6)
	Private insurance	7 (21.9)
	Military/university insurance	4 (12.5)
	No health insurance	8 (25.0)
Usual first point of care for minor symptoms	Community pharmacy	18 (56.3)
	Physician clinic/health center	8 (25.0)
	Home remedies/previous medicines	5 (15.6)
	Emergency department	1 (3.1)
Previous non-prescription antibiotic use	Yes	22 (68.8)
	No	10 (31.2)
Previous use of leftover antibiotics	Yes	14 (43.8)
	No	18 (56.2)
Previous experience of antibiotic refusal	By pharmacist	7 (21.9)
	By physician	6 (18.7)
	No previous refusal experience	19 (59.4)
Reported antibiotic use for child symptoms	Previously requested or used antibiotics for a child	13 (40.6)
	No/not applicable	19 (59.4)

**Table 3:** Analytic themes, core meanings, and main evidence.

Theme	Core Meaning	Main Evidence
Antibiotics as active and responsible care under uncertainty	Antibiotics could signify decisive action, rapid recovery, and responsible caregiving.	Prior prescribing and non-prescription use; child-vignette responses; contrasting preference for monitoring or examination.
Refusal as a negotiation of credibility and care	Credibility depended on whether refusal was accompanied by assessment, explanation, alternatives, and safety-netting.	Reported pharmacist and physician encounters; residual uncertainty; appropriate clinical reassessment.
Continued antibiotic seeking after refusal	Acceptance, reassessment, and continued antibiotic seeking were distinct possible positions.	Reported actions; vignette intentions; observations of others; acceptance-oriented contrasting accounts.

## Data Availability

The data presented in this study are available on request from the corresponding author due to the qualitative nature of the study and the need to protect participant confidentiality. Full interview transcripts and potentially identifiable data are not publicly available. Relevant supporting materials are provided in the article and Supplementary Materials. Further inquiries may be directed to the corresponding author upon reasonable request.

## Supplementary Materials

The following supporting information can be downloaded at https://www.mdpi.com/article/10.3390/healthcare14162612/s1, Supplementary Material S1: COREQ Checklist; Supplementary Material S2: Interview Guide; Supplementary Material S3: Coding Tree and Analytic Development.

## Abbreviations

The following abbreviations are used in this manuscript:

AMR	Antimicrobial resistance
AMS	Antimicrobial stewardship
COREQ	Consolidated Criteria for Reporting Qualitative Research
IRB	Institutional Review Board
KAP	Knowledge, attitudes, and practices
LMICs	Low- and middle-income countries

## References

[1] Murray C.J.L., Ikuta K.S., Sharara F., Swetschinski L., Robles Aguilar G., Gray A., Han C., Bisignano C., Rao P., Wool E. (2022). Global burden of bacterial antimicrobial resistance in 2019: A systematic analysis. Lancet.

[2] Nammi J., Pasala R., Andhe N., Vasam R., Poruri A.D., Sherikar R.R. (2025). Antibiotic Misuse: An In-Depth Examination of Its Global Consequences and Public Health Challenges. Cureus.

[3] Okonkwo R.I., Ndukwe H., Grant G., Khan S. (2025). Antimicrobial stewardship in the community setting: A qualitative exploratory study. Antimicrob. Resist. Infect. Control.

[4] Hedima E.W., Okoro R.N. (2025). Primary health care roles of community pharmacists in low- and middle-income countries: A mixed methods systematic review. BMC Health Serv. Res..

[5] Abed A., Abu Assab M., Merdas Z.J.H., Abu Dayyih W., Maaita M.N., Zakaraya Z., Alotaibi B.S., Alsubaie N. (2026). Confronting antimicrobial resistance in Jordan: Regulatory, economic, and behavioral determinants of non-prescription antibiotic dispensing in community pharmacies—A mixed-methods study. Front. Med..

[6] Almaaytah A., Mukattash T.L., Hajaj J. (2015). Dispensing of non-prescribed antibiotics in Jordan. Patient Prefer. Adherence.

[7] Al-omoush O., Al-Amoosh H., Altal Y., Qasem N., Alshrouf M., Alshawabkeh Y., Souman H., Abu Shamah M., Elfawair H., Karam A. (2025). Dispensing Practice of Non-Prescribed Medications in Community Pharmacies in Jordan: A Simulated Patient Study. Pharm. Pract..

[8] Tanashat M., Albandak M., Al-Ajlouni Y.A., Alqudah K., Manaserh S., Al-Zoubi A.K., Tanashat N.J., Alwarawrah Z., Alziadin N., Batah A.M. (2026). Knowledge, attitudes, and practices regarding antibiotic use among the population of Jordan: A national cross-sectional study. Bayl. Univ. Med. Cent. Proc..

[9] Abdel-Qader D.H., Albassam A., Ismael N.S., El-Shara’ A.A., Shehri A., Almutairi F.S., Al-Harbi D.M., Al Zahrani M.M., Chen L.-C., Al Mazrouei N. (2020). Awareness of Antibiotic Use and Resistance in Jordanian Community. J. Prim. Care Community Health.

[10] Yusef D., Babaa A.I., Bashaireh A.Z., Al-Bawayeh H.H., Al-Rijjal K., Nedal M., Kailani S. (2018). Knowledge, practices & attitude toward antibiotics use and bacterial resistance in Jordan: A cross-sectional study. Infect. Dis. Health.

[11] Ryves R., Eyles C., Moore M., McDermott L., Little P., Leydon G.M. (2016). Understanding the delayed prescribing of antibiotics for respiratory tract infection in primary care: A qualitative analysis. BMJ Open.

[12] Kasse G.E., Humphries J., Cosh S.M., Islam M.S. (2024). Factors contributing to the variation in antibiotic prescribing among primary health care physicians: A systematic review. BMC Prim. Care.

[13] Cabral C., Zhang T., Oliver I., Little P., Yardley L., Lambert H. (2024). Influences on use of antibiotics without prescription by the public in low- and middle-income countries: A systematic review and synthesis of qualitative evidence. JAC Antimicrob. Resist..

[14] Aslam A., Gajdács M., Zin C.S., Ab Rahman N.S., Ahmed S.I., Zafar M.Z., Jamshed S. (2020). Evidence of the Practice of Self-Medication with Antibiotics among the Lay Public in Low- and Middle-Income Countries: A Scoping Review. Antibiotics.

[15] Colliers A., Bombeke K., Philips H., Remmen R., Coenen S., Anthierens S. (2021). Antibiotic Prescribing and Doctor-Patient Communication During Consultations for Respiratory Tract Infections: A Video Observation Study in Out-of-Hours Primary Care. Front Med..

[16] Boiko O., Gulliford M.C., Burgess C. (2020). Revisiting patient expectations and experiences of antibiotics in an era of antimicrobial resistance: Qualitative study. Health Expect..

[17] Gulliford M.C., Charlton J., Boiko O., Winter J.R., Rezel-Potts E., Sun X., Burgess C., McDermott L., Bunce C., Shearer J. (2021). Safety of Reducing Antibiotic Prescribing in Primary Care: A Mixed-Methods Study. Health Soc. Care Deliv. Res..

[18] Bustami M., Alabdali S.A., Assab M.A., Almazari I., Al-Haj W.A., Abu Dayyih W., Alrasheed H.A., Zakaraya Z., Abed A. (2026). Contextual Determinants of Clinical Pharmacists’ Contributions to Team-Based Antimicrobial Stewardship in Jordanian Hospitals: A Realist-Informed Qualitative Study. Antibiotics.

[19] Jordan Center for Disease Control (2024). The National Monitoring & Evaluation Plan for the AMR NAP 2023–2025.

[20] Tong A., Sainsbury P., Craig J. (2007). Consolidated criteria for reporting qualitative research (COREQ): A 32-item checklist for interviews and focus groups. Int. J. Qual. Health Care.

[21] Koopmans E., Schiller D.C. (2022). Understanding Causation in Healthcare: An Introduction to Critical Realism. Qual. Health Res..

[22] Zhang H., Davies C., Su C., O’Donnell D. (2026). Cultural and Structural Influences on Shared Decision-Making in the Care of Critically Ill Patients with Stroke in China: A Critical Realist Qualitative Study. Neurocrit. Care.

[23] Malterud K., Siersma V.D., Guassora A.D. (2016). Sample Size in Qualitative Interview Studies: Guided by Information Power. Qual. Health Res..

[24] Braun V., Clarke V. (2019). Reflecting on reflexive thematic analysis. Qual. Res. Sport Exerc. Health.

[25] Braun V., Clarke V. (2006). Using thematic analysis in psychology. Qual. Res. Psychol..

[26] Braun V., Clarke V. (2021). One size fits all? What counts as quality practice in (reflexive) thematic analysis?. Qual. Res. Psychol..

[27] Torres N.F., Chibi B., Middleton L.E., Solomon V.P., Mashamba-Thompson T.P. (2019). Evidence of factors influencing self-medication with antibiotics in low and middle-income countries: A systematic scoping review. Public Health.

[28] Naser A.Y., Al-Shehri H. (2025). Parental knowledge, attitudes and practices on the use of antibiotics among their children in Jordan: A cross-sectional study. BMJ Paediatr. Open.

[29] Rinaldi A., Bullo A., Schulz P.J. (2025). Patients’ requests and physicians’ prescribing behavior. A systematic review. Patient Educ. Couns..

[30] Li J., Zhou P., Wang J., Li H., Xu H., Meng Y., Ye F., Tan Y., Gong Y., Yin X. (2023). Worldwide dispensing of non-prescription antibiotics in community pharmacies and associated factors: A mixed-methods systematic review. Lancet Infect. Dis..

[31] Alkadhimi A., Dawood O.T., Hassali M.A. (2020). Dispensing of antibiotics in community pharmacy in Iraq: A qualitative study. Pharm. Pract..

[32] Khaddaj R., Salameh P., Al-Hajje A., Bou Dib J., Yeretezian J., Cherfane M., Kotb R., Nakhoul D., Awad R., Iskandar K. (2025). Social determinants of self-medication with leftover antibiotics in Lebanese households: A cross-sectional study. PLoS ONE.

[33] Alhomoud F., Aljamea Z., Almahasnah R., Alkhalifah K., Basalelah L., Alhomoud F.K. (2017). Self-medication and self-prescription with antibiotics in the Middle East-do they really happen? A systematic review of the prevalence, possible reasons, and outcomes. Int. J. Infect. Dis..

[34] Stuart B., Hounkpatin H., Becque T., Yao G., Zhu S., Alonso-Coello P., Altiner A., Arroll B., Böhning D., Bostock J. (2021). Delayed antibiotic prescribing for respiratory tract infections: Individual patient data meta-analysis. BMJ.

