# Supplementary material for: When Antibiotics Are Refused: A Qualitative Study of Patient–Provider Communication, Trust, and Reported or Anticipated Responses in Jordan
Source: Healthcare (Basel). 2026 Aug 19;14(16):2612. doi: 10.3390/healthcare14162612 (PMC13511816; doi:10.3390/healthcare14162612)
Supplement: Supplementary file 1 [file healthcare-14-02612-s001.zip › Supplementary Material S1 COREQ Checklist.pdf]

Supplementary Material S1

Completed COREQ Checklist

When Antibiotics Are Refused: A Qualitative Study of Patient–Provider Communication, Trust, and Reported or Anticipated Responses in Jordan

No.	COREQ item	Guide question	Study-specific response	Location
1	Interviewer/facilitator	Which author(s) conducted the interview or focus group?	AA conducted all one-to-one interviews.	Methods 2.3
2	Credentials	What were the researcher's credentials?	AA was an academic clinical pharmacist with postgraduate research training.	Methods 2.3
3	Occupation	What was the researcher's occupation at the time of the study?	Academic clinical pharmacist.	Methods 2.3
4	Gender	Was the researcher male or female?	Male.	Methods 2.3
5	Experience and training	What experience or training did the researcher have?	Previous qualitative interviewing experience and postgraduate research training; the wider team had experience in pharmacy practice, antimicrobial stewardship, public health, and qualitative research.	Methods 2.3
6	Relationship established	Was a relationship established before study commencement?	No prior personal or professional relationship existed between the interviewer and participants.	Methods 2.3
7	Participant knowledge of the interviewer	What did participants know about the researcher?	Participants received an information sheet describing the study purpose and interview procedures and were informed that participation was voluntary. The interviewer had no prior relationship with participants.	Methods 2.6 and 2.11
8	Interviewer characteristics	What interviewer characteristics were reported?	The interviewer was a male academic clinical pharmacist. Reflexive attention was given to the possibility that the team's pharmacy and stewardship backgrounds could privilege professional interpretations; neutral prompts were used and disagreement, frustration, and distrust were invited.	Methods 2.3
9	Methodological orientation and theory	What methodological orientation was stated?	Experiential reflexive thematic analysis situated within a contextualist, critical-realist position; coding was primarily semantic with selective latent interpretation.	Methods 2.1 and 2.9
10	Sampling	How were participants selected?	Purposive maximum-variation sampling, deliberately enriched for relevant antibiotic-related experience.	Methods 2.5
11	Method of approach	How were participants approached?	Standardized invitations were circulated through WhatsApp, Facebook, community networks, and non-healthcare contacts. Interested individuals self-referred through Google Forms, telephone, or WhatsApp. Invitations could be forwarded to acquaintances, but participation remained voluntary.	Methods 2.6
12	Sample size	How many participants were in the study?	Thirty-two adults.	Results 3.1; Methods 2.5
13	Non-participation	How many people refused or dropped out, and why?	Forty-three expressed interest; 39 completed screening. Reasons for four not completing screening were not recorded. Three screened individuals were ineligible. Of 36 eligible invitees, 32 completed interviews; two could not be scheduled, one cancelled because of time constraints, and one was lost to follow-up. No participant withdrew after an interview began.	Methods 2.5
14	Setting of data collection	Where were data collected?	Remote telephone or online video interviews in Jordan. Participants confirmed that they were in a private setting before the interview.	Methods 2.2
15	Presence of non-participants	Was anyone else present besides participants and researchers?	Participants confirmed a private setting where they could speak freely and without interruption; no non-participants were reported as present.	Methods 2.2

No.	COREQ item	Guide question	Study-specific response	Location
16	Description of sample	What important sample characteristics were reported?	Age, gender, region and type of residence, education, employment, marital and caregiving status, insurance, usual first point of care, previous non-prescription antibiotic use, leftover use, prior refusal, and child-related antibiotic use.	Results 3.1 and Table 2
17	Interview guide	Were questions/prompts/guides provided and pilot tested?	A semi-structured guide was used. Personal narratives were elicited before four vignettes. The guide was piloted with three adults; pilot interviews were excluded after minor wording revisions. The complete guide is provided in Supplementary Material S2.	Methods 2.7; Table 1; Supplement S2
18	Repeat interviews	Were repeat interviews conducted?	No.	Methods 2.7
19	Audio/visual recording	Was audio or visual recording used?	All interviews were audio-recorded with participant permission.	Methods 2.7 and 2.11
20	Field notes	Were field notes made?	Yes. Field notes were completed immediately after each interview and used for contextual observations, familiarization, initial analytic impressions, and later probing; they were not analyzed as an independent dataset.	Methods 2.7 and 2.10
21	Duration	What was the duration of interviews?	Approximately 30–45 minutes.	Methods 2.7
22	Data saturation	Was data saturation discussed?	The study did not claim complete thematic saturation. Sample sufficiency was assessed using information power, considering the focused aim, participant relevance and diversity, depth of dialogue, and adequacy for an interpretive account.	Methods 2.5
23	Transcripts returned	Were transcripts returned to participants?	No full transcripts were returned for correction or comment.	Methods 2.10
24	Number of data coders	How many researchers coded the data?	AA led coding and theme development. A second researcher read six early transcripts and engaged in collaborative reflexive discussion; the wider team discussed developing themes and deviant cases. This was not consensus coding or independent verification.	Methods 2.9
25	Description of coding tree	Was a coding tree described?	An evolving thematic map and analytic development record documented code families, central organizing concepts, evidence-source distinctions, and theme refinement. It did not function as a fixed codebook.	Methods 2.9; Supplement S3
26	Derivation of themes	Were themes identified in advance or derived from data?	Themes were predominantly data-led but sensitized by the interview guide and literature on antibiotic expectations, trust, healthcare access, and stewardship. Early descriptive topic clusters were refined into three analytic themes.	Methods 2.9; Supplement S3
27	Software	What software was used?	NVivo 14 supported data organization and retrieval.	Methods 2.9
28	Participant checking	Did participants provide feedback on findings?	A brief Arabic summary of preliminary findings was shared with six participants who agreed to further contact. Feedback was used to assess resonance, clarity, and comprehensibility, not to validate a single objectively correct interpretation.	Methods 2.10
29	Quotations presented	Were participant quotations used to illustrate themes?	Yes. Contextually informative extracts were identified by participant code and limited relevant characteristics, with the evidence source stated as personal experience, vignette response, observation of others, or generalized perception.	Results 2.2–2.6; Table 2; Supplement S4
30	Data and findings consistent	Was there consistency between the data presented and the findings?	The narrative interpretation is linked to extracts and explicitly distinguishes reported behavior from anticipated and socially attributed possibilities.	Results 3.2–3.6; Table 3
31	Clarity of major themes	Were major themes clearly presented?	Three central themes were presented and defined by their organizing concepts: antibiotics as active and responsible care under uncertainty; refusal as a negotiation of credibility and care; and continued antibiotic seeking after refusal as reported, anticipated, and socially attributed possibilities.	Results 3.3–3.5; Supplement S3
32	Clarity of minor themes and diverse cases	Were diverse or minor cases discussed?	Contrasting and deviant accounts included acceptance without antibiotics, persistent uncertainty, legitimate reassessment, continued seeking, and variation by prior refusal experience, non-prescription use, parenting, healthcare access, region, and education where supported.	Results 3.3–3.6; Supplement S3
