# Supplementary material for: When Antibiotics Are Refused: A Qualitative Study of Patient–Provider Communication, Trust, and Reported or Anticipated Responses in Jordan
Source: Healthcare (Basel). 2026 Aug 19;14(16):2612. doi: 10.3390/healthcare14162612 (PMC13511816; doi:10.3390/healthcare14162612)
Supplement: Supplementary file 1 [file healthcare-14-02612-s001.zip › Supplementary Material S2 Interview Guide.pdf]

Supplementary Material S2

Semi-Structured Interview Guide

When Antibiotics Are Refused: A Qualitative Study of Patient–Provider Communication, Trust, and Reported or Anticipated Responses in Jordan

English-language reporting version of the Arabic guide used for the study. Interviews were conducted in Arabic.

Purpose and use of the guide

This guide was designed to explore adults' experiences, interpretations, and anticipated responses surrounding antibiotic use and antibiotic refusal by pharmacists and physicians in Jordan. The interview sequence prioritized spontaneous personal narratives before vignette prompting. The guide was applied flexibly: probes were selected according to each participant's experience, and not every probe was asked verbatim.

Interviewer instruction: use neutral, non-judgmental prompts; do not correct participants or provide stewardship education during the interview. Where participants ask for clinical advice, explain that the interview is for research and advise them to seek an appropriate healthcare professional.

A. Pre-interview procedures

1. Confirm eligibility and that the participant has received or had explained the participant information sheet.
2. Confirm that the participant is in a private setting and can speak freely without interruption.
3. Answer questions about the study.
4. Obtain recorded verbal consent for participation, audio recording, and use of anonymized quotations; document consent in the dated consent log.
5. Remind the participant that participation is voluntary, any question may be skipped, and the interview may be stopped at any time.

B. Opening script

"Thank you for taking part. We are interested in how members of the public think about antibiotics and how they respond when a pharmacist or physician advises that an antibiotic is not needed. There are no right or wrong answers. We are interested in your own experiences and views, including disagreement, uncertainty, or frustration. I will first ask about your experiences. Later, I will present several hypothetical situations. Please tell me what you think you might do in those situations."

C. Healthcare seeking and recent illness experiences

1. When you or someone in your household develops a minor acute illness, such as a sore throat, cough, fever, or cold symptoms, what do you usually do first?

- Why is that usually your first choice?
- How do cost, distance, waiting time, work, family responsibilities, or insurance influence that choice?
- When would you go directly to a physician or health center?

2. Please tell me about the most recent time you used an antibiotic.

- What symptoms did you have?
- Who recommended or supplied it?
- Was it prescribed, obtained from a pharmacy without a prescription, or already available at home?
- Did you ask for a specific antibiotic by name? Why?
- What happened after you took it?

3. How do you usually decide whether an illness needs an antibiotic?

- What makes an illness seem severe or "strong"?
- Do previous prescriptions influence your decision?

- What role do family, friends, or social media play?
- Does your decision change when the patient is a child?

D. Spontaneous personal experience of antibiotic refusal

Ask this section before introducing any vignette. If the participant has experienced more than one refusal, explore the clearest or most recent encounter and distinguish pharmacist from physician encounters.

4. Have you ever expected, requested, or asked about an antibiotic and been told by a pharmacist or physician that it was not needed or would not be provided?

- Please tell me what happened from the beginning.
- Who refused: a pharmacist or a physician?
- What symptoms or concerns did you have?
- What did the provider ask, examine, explain, or recommend?
- How did you feel at that moment?
- What did you understand the reason for refusal to be?

5. What did you do after that encounter?

- Did you accept the advice and monitor your symptoms?
- Did you use symptomatic treatment?
- Did you seek medical reassessment? What was your purpose?
- Did you visit another pharmacy or provider mainly to obtain an antibiotic?
- Did you use an antibiotic stored at home or provided by someone else?
- What was the eventual outcome?

6. Looking back, what made the refusal easier or harder to accept?

- Previous prescriptions for similar symptoms?
- Provider confidence, empathy, or explanation?
- Symptom severity or persistence?
- Access, cost, transport, work, or caregiving responsibilities?

E. Trust, professional roles, and meanings of care

7. What makes you trust advice that an antibiotic is not needed?

- What would show that your illness had been taken seriously?
- How important are symptom questions, examination, explanation, alternatives, and warning signs?

8. Do you interpret the same advice differently when it comes from a pharmacist rather than a physician?

- What do you see as the role or authority of each professional?
- Would you seek confirmation from another provider? Why?

9. What does receiving an antibiotic represent to you?

- Strong treatment? Faster recovery? Returning to work or responsibilities? Responsible care for a child?
- Can waiting, symptom management, or reassessment also feel like active care?

F. Vignettes

Introduce each vignette as hypothetical. Responses should be treated as anticipated behavior, not as documented personal action. Follow the participant’s reasoning and avoid implying that a diagnosis can be made from nonspecific symptoms.

Vignette 1: Adult/pharmacist

“Imagine that you have had sore throat, cough, runny nose, and mild fever for two days. You go to a community pharmacy and ask for an antibiotic. The pharmacist says that antibiotics are not needed and recommends symptomatic treatment instead. How would you feel? Would you accept the pharmacist’s advice? What would you do next?”

Optional probes:

- What would make you accept or question the advice?
- Would you wait, seek medical assessment, try another pharmacy, or use medicine already at home?
- What explanation or follow-up advice would you need?

Interpretive caution: The symptoms are nonspecific. The vignette explores interpretation and anticipated response, not clinical diagnosis.

Vignette 2: Child/pharmacist

"Imagine that your child has sore throat, cough, and fever for two days. The pharmacist says antibiotics are not appropriate now and advises fever control, fluids, and medical review if symptoms worsen or continue. Would your response be different because the patient is a child?"

Optional probes:

- What concerns you most when a child has these symptoms?
- What information about fever, warning signs, expected duration, or when to see a physician would help?
- Would your response differ according to the child's age or health?

Interpretive caution: The child's age, comorbidities, and risk profile were not specified; participants may imagine different pediatric contexts.

Vignette 3: Previous prescription

"Imagine that you had similar symptoms last year and a doctor prescribed an antibiotic. This time, a pharmacist or physician says that antibiotics are not needed. How would you interpret this difference?"

Optional probes:

- Which experience would you trust more and why?
- Would the previous prescription make refusal seem inconsistent?
- What explanation could reconcile the two encounters?

Interpretive caution: The vignette examines the influence of prior prescribing. Pharmacist and physician refusal are combined and should not be interpreted as a controlled comparison.

Vignette 4: Communication contrast

Provider A says: "No, you do not need antibiotics." Provider B acknowledges the wish to recover, explains that antibiotics are unlikely to help because the illness appears viral, recommends symptomatic treatment, and gives warning signs and advice about when to seek further care. "Which response would you trust more, and why?"

Optional probes:

- Which part of the response mattered most?
- Would the detailed response change what you might do next?
- Would you still feel uncertain?
- What wording would feel respectful and credible?

Interpretive caution: The detailed response varies several components simultaneously—empathy, explanation, a viral interpretation, alternatives, and safety-netting—so any preference cannot be attributed to one component.

G. Possible routes after refusal and perceived community norms

10. What different things might people do after being told that an antibiotic is not needed?

- Try another pharmacy?
- Seek a physician for assessment or mainly for a prescription?
- Use leftover or shared antibiotics?
- Ask for a named antibiotic?
- Repeat or emphasize symptoms?

11. Which of these have you personally done, which have you observed in someone else, and which are only general possibilities?

- Ask explicitly for the source of each account.
- Clarify whether seeking another clinician was for reassessment or to obtain a predetermined antibiotic.

12. Why might one person accept refusal while another continues seeking an antibiotic?

- Previous benefit? Trust? Child illness? Cost, access, or time? Inconsistent provider advice?

H. Stewardship communication preferences

13. What should a pharmacist or physician say or do when an antibiotic is not recommended?

- Acknowledge concern? Explain why? Offer symptom relief? Explain expected duration? Give warning signs and follow-up advice?

14. Which messages are most meaningful to you?

- Personal side effects or harm? Antibiotics not working later? Risk to a child or family? Wider antimicrobial resistance?

15. What advice would you give pharmacists, physicians, or public-health campaigns about antibiotic refusal?

I. Closing

16. Is there anything else about antibiotics, refusal, trust, or seeking care that you think is important and that we have not discussed?

Thank the participant, remind them that their account will be anonymized, and stop the audio recording.

J. Post-interview field-note and indexing prompts

- Interview mode and duration; confirmation of private setting; interruptions or technical issues.
- Key emotional or interactional features, hesitation, contradiction, or uncertainty.
- Potential influence of interviewer wording or vignette sequence.
- Issues to probe in subsequent interviews.
- Evidence-source tag for each relevant extract: EXP (personal experience), VIG (vignette-elicited anticipated response), OBS (observation of another person), or GEN (generalized view).
- Context tags where analytically relevant: provider type, prior refusal, prior non-prescription use, leftover access, caregiving status, healthcare access, region, education, stated purpose of further care, and reported outcome.
