# Supplementary material for: When Antibiotics Are Refused: A Qualitative Study of Patient–Provider Communication, Trust, and Reported or Anticipated Responses in Jordan
Source: Healthcare (Basel). 2026 Aug 19;14(16):2612. doi: 10.3390/healthcare14162612 (PMC13511816; doi:10.3390/healthcare14162612)
Supplement: Supplementary file 1 [file healthcare-14-02612-s001.zip › Supplementary Material S3 Coding Tree and Analytic Development.pdf]

Supplementary Material S3

Evolving Thematic Map, Coding Tree, and Analytic Development Record

When Antibiotics Are Refused: A Qualitative Study of Patient–Provider Communication, Trust, and Reported or Anticipated Responses in Jordan

Methodological note

The analysis used an experiential form of reflexive thematic analysis within a contextualist, critical-realist orientation. Coding was predominantly data-led but sensitized by the interview guide and literature. Codes were treated as interpretive tools rather than objective units requiring coder agreement. AA led coding and theme development; a second researcher read six early transcripts and contributed collaborative reflexive discussion, and the wider team discussed developing themes and contrasting cases. The structure below documents the final relationship between evidence-source indexing, code families, and central organizing concepts.

Cross-cutting evidence-source indexing

Index	Definition	Permitted reporting language	Example analytic use
EXP – Personal experience	A personally recalled event, action, or outcome involving the participant.	“reported,” “described having,” or “personally experienced.”	Strongest evidence for what occurred after an actual refusal.
VIG – Vignette response	An anticipated response to a hypothetical scenario introduced by the interviewer.	“anticipated,” “said they might,” or “in the vignette.”	Explores conditional intentions; not reported as actual behavior.
OBS – Observation of others	An account of a relative, friend, or community member’s behavior.	“observed,” “described another person,” or “perceived family practice.”	Illuminates social norms without attributing the action to the participant.
GEN – Generalized view	A broad statement about what “people” might do, without a specific observed case.	“perceived,” “suggested,” or “believed people may.”	Used cautiously as a perception, not behavioral evidence.

Contextual attributes were recorded where analytically meaningful: prior refusal experience, provider type, previous non-prescription use, leftover availability, caregiving status, healthcare access, region, education, stated purpose of further care, and reported outcome. These attributes informed comparison but were not treated as deterministic subgroup variables.

Theme 1: Antibiotics as active and responsible care under uncertainty

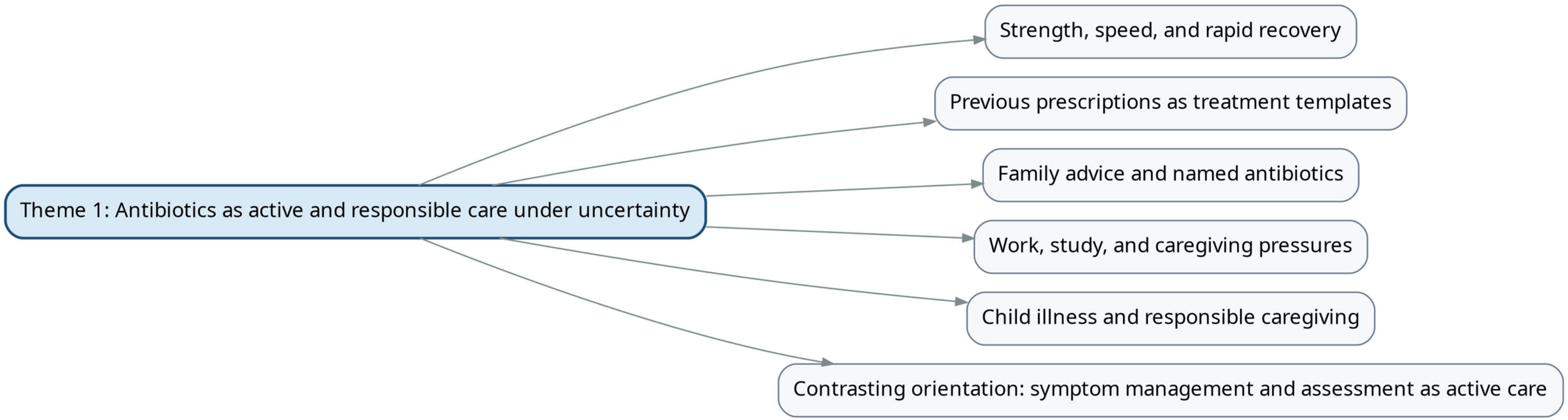

Figure S1. Code families supporting theme 1: antibiotics as active and responsible care under uncertainty. Branches are interpretive and may overlap; they are not mutually exclusive categories.

Theme 2: Refusal as a negotiation of credibility and care

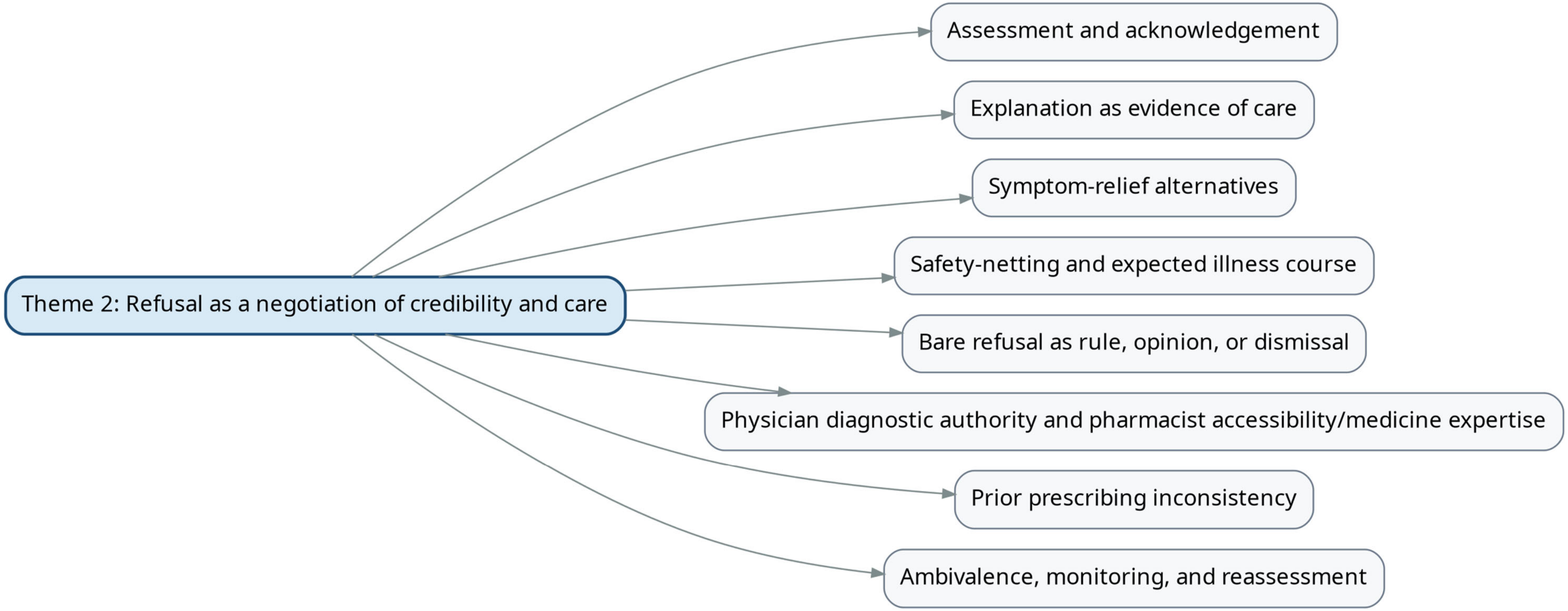

Figure S3.2. Code families supporting theme 2: refusal as a negotiation of credibility and care. Branches are interpretive and may overlap; they are not mutually exclusive categories.

Theme 3: Continued antibiotic seeking after refusal: reported, anticipated, and socially attributed possibilities

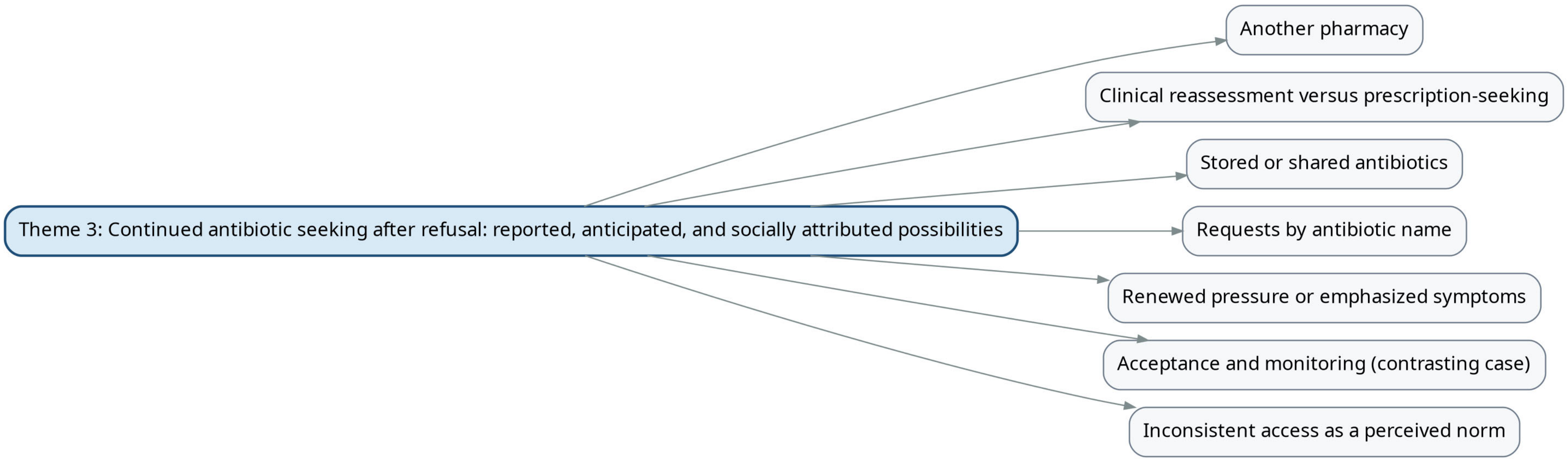

Figure S3.3. Code families supporting theme 3: continued antibiotic seeking after refusal: reported, anticipated, and socially attributed possibilities. Branches are interpretive and may overlap; they are not mutually exclusive categories.

Theme-level analytic structure

Final theme	Central organizing concept	Main code families	Selective latent meanings	Contrasting/deviant accounts
1. Antibiotics as active and responsible care under uncertainty	Antibiotics could function as a practical and symbolic response to uncertainty, disruption, and caregiving responsibility, not only as a treatment believed to cure all infections.	Strength/rapid recovery; return to work or study; previous prescriptions as templates; family advice and remembered names; symptom severity; child illness; fear of deterioration.	Decisive action; control under uncertainty; responsibility; "strong" care.	Participants who preferred symptom management, monitoring, or clinical assessment and did not equate antibiotics with stronger care.
2. Refusal as a negotiation of credibility and care	Refusal was interpreted through evidence that the provider had taken the illness seriously and offered an active care plan.	Acknowledgement; symptom questions or examination; reasoned explanation; symptom-relief alternatives; expected duration; warning signs; empathy; bare refusal; provider authority; inconsistency with prior prescriptions; residual doubt.	Credibility; recognition; professional authority; relational care; dismissal.	Participants who understood the explanation but remained uncertain; those who sought appropriate reassessment rather than a willing antibiotic supplier.
3. Continued antibiotic seeking after refusal: reported, anticipated, and socially attributed possibilities	Refusal could be treated as definitive care, a conditional recommendation, or a negotiable/local barrier; the meaning depended on evidence source and stated purpose.	Another pharmacy; physician consultation; reassessment versus prescription-seeking; leftovers; family sharing; named requests; renewed pressure; emphasized symptoms; acceptance and monitoring; inconsistent dispensing as a norm.	Negotiability of refusal; accessibility; continuity versus fragmentation of professional standards.	Acceptance without further seeking; clinical reassessment for persistent symptoms; hypothetical or third-person accounts not treated as personal behavior.

Analytic development record

Early descriptive cluster	Reflexive analytic question	Refinement decision	Final location
Antibiotics as "strong treatment," rapid recovery, previous prescriptions, family advice, and child illness	What common meaning links these topics beyond misconceptions about bacterial versus viral illness?	Integrated as antibiotics representing active and responsible care under uncertainty, while retaining contrasting orientations toward monitoring and assessment.	Theme 1
Refusal as a test of trust	What is being evaluated when a participant accepts, questions, or rejects refusal?	Reframed around professional credibility and whether the illness was taken seriously.	Theme 2
Communication that converts refusal into care	Does communication form a separate theme, or does it explain how refusal acquires meaning?	Merged with trust because explanation, alternatives, and safety-netting functioned as evidence of care rather than a separate behavioral mechanism. Causal wording such as "converts" was removed.	Theme 2
Antibiotic shopping after refusal	Which accounts document personal action, and which reflect intentions or perceived social norms?	Renamed "continued antibiotic seeking after refusal" and indexed by personal experience, vignette response, observation of others, or generalized view.	Theme 3

Early descriptive cluster	Reflexive analytic question	Refinement decision	Final location
		Legitimate reassessment was separated from prescription-seeking.	
Linear behavioral pathway	Do participant accounts establish a stable, sequential, causal pathway?	Replaced with a non-causal interpretive map allowing movement among acceptance, uncertainty/reassessment, and continued seeking across providers and illness episodes.	Figure 1 / Section 3.7
Demographic comparisons	Do age, education, region, parenting, prior use, or access form coherent explanatory patterns?	Reported only where several accounts supported a plausible contextual pattern; no statistical or deterministic subgroup claims were made.	Section 3.6
